# Anal function and quality of life analysis after laparoscopic modified Parks for ultra-low rectal cancer patients

**DOI:** 10.1186/s12957-020-1801-7

**Published:** 2020-02-03

**Authors:** Haibo Ding, Jian Li, Yuxiang Chen, Zhi Yang, Zha Peng, Xin Liao

**Affiliations:** 1 Hepatobiliary and Enteric Surgery Research Center, Xiangya Hospital, Central South University, 87 Xiangya Road, Changsha, 410008 Hunan China; 2 School of Pharmaceutical Science, Central South University, 172 Tongzip Road, Changsha, 410013 Hunan China

**Keywords:** Modified transanal coloanal anastomosis (Parks surgery), FIQL, Anal function, Ultra-low position, Rectal cancer, Anus-preserving surgery

## Abstract

**Background:**

To assess postoperative anal function and quality of life of ultra-low rectal cancer patients treated by laparoscopic modified Parks surgery.

**Methods:**

From February 2017 to March 2019, 114 patients with ultra-low rectal cancer above T2 were treated respectively with ultra-low anterior resection (Dixon), modified coloanal anastomosis (modified Parks), and Miles according to the preoperative stage and anastomotic position. The postoperative anal function and Fecal Incontinence Quality of Life Scale (FIQL) of each patient were collected and synthetically analyzed.

**Results:**

Compared with the Dixon group, the postoperative anal function and FIQL in the Parks group were poor at the early stage. However, from 6 to 12 months after surgery, the scores of anal function and FIQL in the Parks group were similar to those in the Dixon group (*P* > 0.05). Compared with the Miles group, the FIQL of the two groups were similar in the early postoperative stage. However, with the passage of time, from 3 to 9 months after surgery, the four domains of FIQL in the Parks group were higher than those in the Miles group successively (*P* < 0.05).

**Conclusions:**

Laparoscopic modified Parks is a safe, effective, and economical anus-preserving surgery. Although its early anal function and FIQL were poor, it could gradually recover to the similar level as Dixon. Moreover, it can save the anus and obtain a better postoperative quality of life for some patients who previously could only undergo Miles.

## Background

Traditionally, in order to ensure radical treatment, for ultra-low rectal cancer within 5 cm from the lower edge of the tumor to the anus, it is considered that combined abdominal and perineal resection (APR), that is, Miles surgery, is the standard treatment [1]. However, permanent stoma also brings more stoma-related complications [2] and urogenital dysfunction [3, 4] to patients. In addition, long-term nursing care of stoma [5], persistent mental pressure of stoma on patient’s image change, and other factors seriously affect the quality of life [6] and the realization of social function [7] of patients after Miles, making patients’ attitude toward the postoperative treatment more negative. Therefore, many patients with rectal cancer have a strong sense of rejection of stoma before surgery [8]. With the development of preoperative neoadjuvant radiotherapy and chemotherapy [9, 10] and laparoscopic techniques [11], as well as the confirmation of the principle of total mesorectal excision (TME) [12–15] and the understanding of the concept of circumferential margin [16], the prognosis of anus-conserving surgery has been greatly improved. There are also more and more related research and application of anus-conserving surgery. However, there is still controversy about whether anus-preserving surgery should be performed for ultra-low rectal cancer. The focus is whether the radical resection of the tumors and a good anal function can be guaranteed after the resection of all or part of the internal sphincter [17].

The key to the radical treatment of anus-preserving surgery is to ensure the safety of distal incision margin, which has been a research hot spot for a long time [18–21]. Many related literatures have confirmed that 1 cm distal resection margin does not affect the oncological safety of rectal cancer [21, 22]. These studies and findings provide a theoretical basis for expanding the indications of anus-preserving surgery for ultra-low rectal cancer. However, there are few studies on anal function and quality of life after anus-preserving surgery, and most of them focus on patients undergoing intersphincteric resection (ISR) [23] and adjuvant radiotherapy and chemotherapy after ISR [24].

By combining the advantages of traditional Bacon surgery [25, 26], the modified Parks surgery was used in the Department of Colorectal and Anal Surgery of Xiangya Hospital of Central South University, China, to perform anus-preserving surgery for ultra-low rectal cancer, and the anal function and FIQL of the patients after modified Parks surgery were further studied. The results showed that the modified Parks surgery could achieve a good anal function and FIQL for patients with ultra-low rectal cancer while preserving the anus.

## Methods

### Patient data

A total of 114 patients with ultra-low rectal cancer above T2 were recruited, excluding those with preoperative anal laxity on rectal examination; Williams anal function rating above B; serious heart, brain, and lung diseases; and abdominal and pelvic operation history. After admission, the patients’ condition was evaluated comprehensively, and the preoperative staging was carried out according to the results of preoperative enteroscopy, enhanced CT, MRI, and intraoperative conditions. According to the stage of the patients and the position of the anastomotic opening after the distal disconnection, the patients with the anastomotic opening at or below the dentate line underwent laparoscopic modified Parks operation, the patients with the anastomotic opening above the dentate line but about 2 cm away from the dentate line underwent laparoscopic ultra-low Dixon operation, the patients with the invasion or distant metastasis of the sphincter or pelvic organs could not undergo radical resection under laparoscope Miles operation, and the patients were divided into three groups. All patients in this study gave informed consent. The patient was told that the operation would be performed under laparoscope, the operative type would be decided by the same surgeon, and the operation would be converted to open operation if necessary. There were no significant differences in age and sex among the three groups (*P* > 0.05), with clinical comparability (Table 2).

### Surgical techniques

**Fig. 1:**
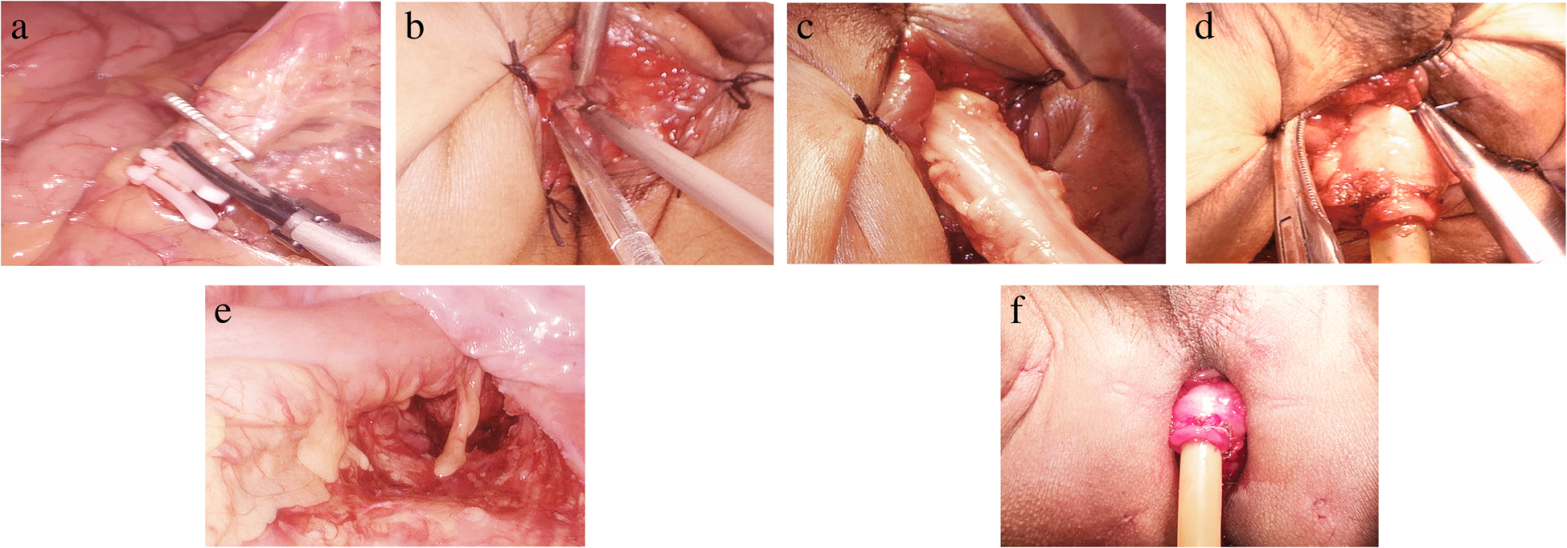
One-stage operation. **a** Transection of submesenteric vessels. **b** Operation of perineal intestine. **c** Towed distal intestinal tube. **d** Colon and anus anastomosis. **e** Intraabdominal intestinal tube after anastomosis. **f** Perineal intestine indwelling anal canal after operation

After general anesthesia intubation, the patients took the improved lithotomy position, and the operation was divided into abdominal operation and perineal operation (Fig. 1).

#### Abdominal operation

The vessels were cut off at the root of the inferior mesenteric artery, and lymph nodes were dissected. The mesentery was separated under the TME principle. The sacro rectal ligament and part of levator ani muscles were cut off to reach the upper edge of the external anal sphincter and dentate line. Some patients continued to free 1–2 cm downward through sphincter space.

#### Perineal operation

At first, the mucosa and internal sphincter were incised vertically in the predetermined edge which is 1–2 cm below the tumor. Next, we moved upward from the gap of the sphincter to communicate with the pelvis cavity and dragged the colon out of the body through the anus. Then, we trimmed the mesentery and amputated the colon at 10 cm above the tumor and preserved 3–5 cm intestinal canal outside the anus. Pathological examination of frozen sections during the operation was performed to ensure that the margin of incision was negative, and Miles was performed for positive cases. The colon stump was repaired, and hemostasis was thoroughly carried out. The seromuscular layer of the intestinal wall and the cutting edge of anal margin skin was sutured discontinuously with 3–0 absorbable suture according to four quadrants to ensure no bleeding at the anastomotic site. The front of the anal canal was wrapped with 5-cm-long Vaseline gauze to compress hemostasis and drain excrement. Finally, we reconstructed the pneumoperitoneum and examined the pelvic active bleeding and anastomotic tension by laparoscopy.

**Fig. 2:**
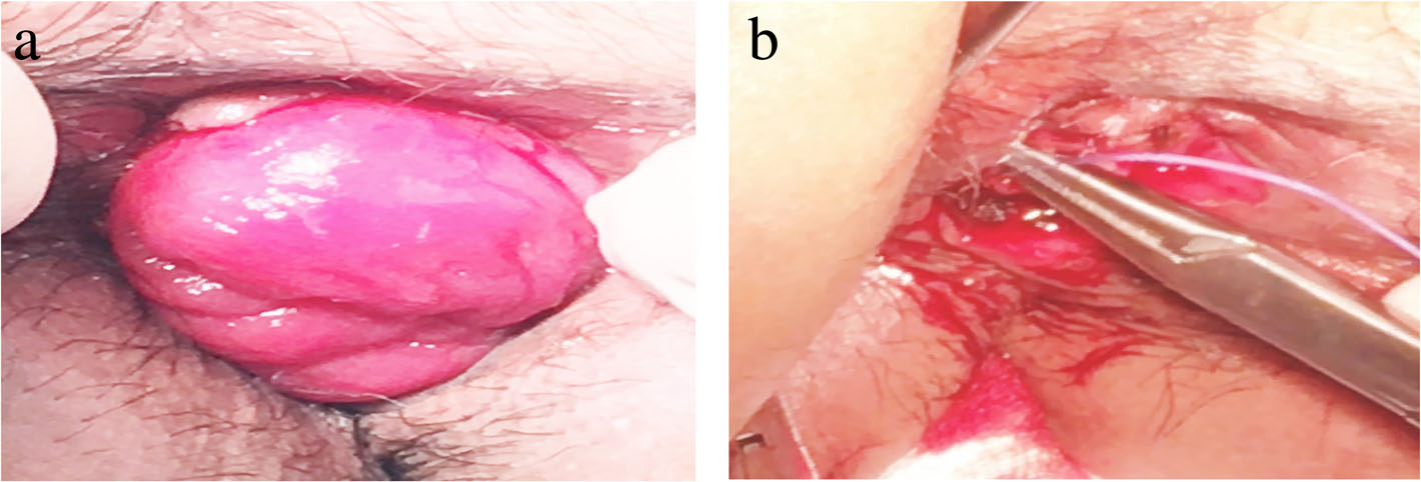
Two-stage operation. **a** External intestinal canal with good blood supply. **b** Repair of stump after excision of external intestinal tube

The blood supply of external intestinal canal was observed after operation in the modified Parks group. As for some patients, due to sphincter contraction and other reasons, ischemic atrophic external intestinal tube can be cut off. For patients with good blood supply of external intestinal canal, external intestinal canal resection can be performed at the edge of the anus when they return to the hospital for the first review about 14 days after surgery (Fig. 2). One week after the operation, all the patients who kept their anus were instructed to perform anal contraction exercise four to six times a day for 15–30 min each time, to keep the anus clean, and to have warm water sitting bath. Rectal examination was performed 1 month after operation. If it is difficult for the anus to pass through the index finger, the anus should be dilated regularly. According to the TNM stage of colorectal cancer of the American Cancer Committee on Cancer (AJCC), patients with stage II pathological stage and high-risk factors or stage III patients were treated with six to eight courses of chemotherapy with mFOLFOX6 regimen. The patients with poor pathological type and late stage received synchronous chemotherapy.

### Data collection and postoperative follow-up

The clinical data of patients were collected. The anal function indexes of patients were followed up in 1, 3, 6, 9, and 12 months after surgery, including Williams classification standard, LARS score, Cleveland Clinic Florida FI score (Wexner scale), and Fecal Incontinence Quality of Life Scale (FIQL).

### Statistical analysis

SPSS 23.0 statistical software was used for statistical analysis. The counting data were expressed by the rate (%), and the normal distribution measurement data were expressed by means ± SD, and those data were all accurate to the last decimal point. *χ*^2^ test or Fisher exact probability method was used to compare the counting data between groups; *t* test was used to compare the FIQL and other measurement data between the two groups; four-grid *χ*^2^ test was used to compare the anal function rating. *p* < 0.05 was considered to indicate a statistically significant difference between the data sets.

## Results

In this study, all patients were operated successfully, the mesorectal excision was complete, and there was no rectal rupture. Pathological sections during and after surgery showed that both margins were negative. The basic data, *t* test results, and postoperative pathological results of the three groups were collected (Tables 1, 2, 3, and 4). All the patients did not take other exercises and treatment except for postoperative anal function rehabilitation guidance. One patient in the Parks group had fecal fluid drained from the vagina after operation, which was confirmed as rectovaginal fistula by postoperative radiography. Six patients in the Parks group had fecal fluid drained from abdominal drainage tube after operation, which was confirmed as anastomotic leakage by postoperative radiography. After the conservative treatment was ineffective, these seven patients underwent terminal ileostomy and were excluded from postoperative follow-up.

**Table 1:** Basic information of patients in the three groups

Groups	Number	Gender		Age (years)	BMI	Total hospitalization cost (Yuan)	Distance from lower tumor edge to anal margin (cm)
Parks	49	Male	25	55.4 ± 11.7	22.9 ± 3.0	53,052.9 ± 8021.8	2.8 ± 1.1
		Female	24				
Dixon	44	Male	30	57.8 ± 11.5	22.3 ± 2.6	58,398.4 ± 12,198.2	2.3 ± 1.2
		Female	14				
Miles	21	Male	12	56.4 ± 9.3	21.6 ± 2.9	61,584.2 ± 9776.6	4.3 ± 0.8
		Female	9				

Data are presented as mean ± SD

**Table 2:** *T* test results of basic information in the three groups

Groups	Gender	Age	BMI	Total hospitalization cost	Tumor distance from anal margin
Parks-Dixon	*t* = 0.009	*t* = 0.927	*t* = 0.412	*t* = 0.057	*t* = 0.120
	*P* = 0.095	*P* = 0.317	*P* = 0.030	*P* = 0.000	*P* = 0.000
Parks-Miles	*t* = 0.464	*t* = 0.241	*t* = 0.911	*t* = 0.151	*t* = 1.655
	*P* = 0.644	*P* = 0.727	*P* = 0.366	*P* = 0.016	*P* = 0.102

**Table 3:** *χ*^2^ test results of Williams classification standard of Parks-Dixon group

Groups	One months after surgery	Three months after surgery	Six months after surgery	Nine months after surgery	Twelve months after surgery
Parks-Dixon group	Pearson *χ*^2^ = 19.35	Pearson *χ*^2^ = 7.13	Pearson *χ*^2^ = 1.62	Pearson *χ*^2^ = 0.80	Pearson *χ*^2^ = 0.00
	*P* = 0.000	*P* = 0.008	*P* = 0.203	*P* = 0.371	*P* = 1.000

**Table 4:** Pathological results of the three groups

Groups	Degree of differentiation			TNM stage				
	Poor	Moderate	High	0	I	II	III	IV
Parks	3	37	9	0	11	20	18	0
Dixon	9	28	7	0	9	9	26	0
Miles	11	8	2	0	3	4	10	4

Postoperative Fecal Incontinence Quality of Life Scale (FIQL) was divided into four domains: lifestyle, coping/behavior, depression/self-perception, and embarrassment. After 1, 3, 6, 9, and 12 months of surgery, the scores of each domain in each group were collected, and their average values were taken (Fig. 5).

### Parks-Dixon group

#### Postoperative anal function

##### Williams classification standard

**Fig. 3:**
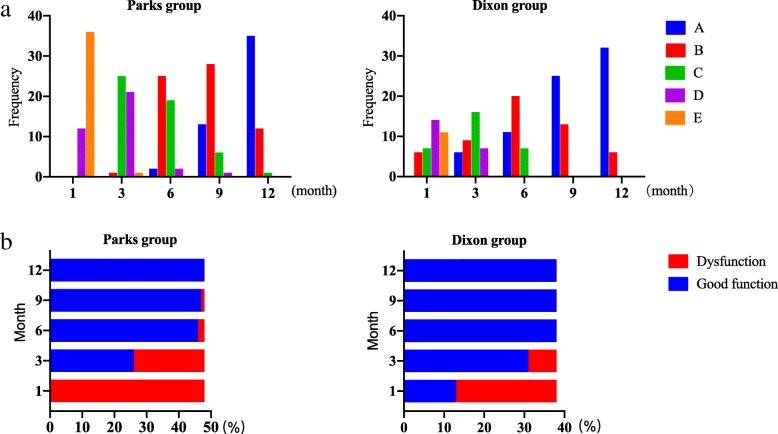
Williams anal function rating. **a** Frequency distribution of Williams anal function rating in Parks group and Dixon group. **b** The rate of good anal function and dysfunction of Williams in the Parks group and Dixon group. A, B, and C indicate good function. D and E indicate dysfunction

From 1 to 3 months after surgery, the anal function of the Parks group was poor (P1 = 0.000, P3 = 0.008; good rate 0.00–34.21% vs 54.20–81.58%), but from 6 months after surgery, the anal function of the two groups was similar (P6 = 0.203; good rate 95.80% vs 100%); until 12 months after surgery, the good rate of the two groups reached 100% (Table 3, Figs. 3b and 6).

##### LARS score

**Fig. 4:**
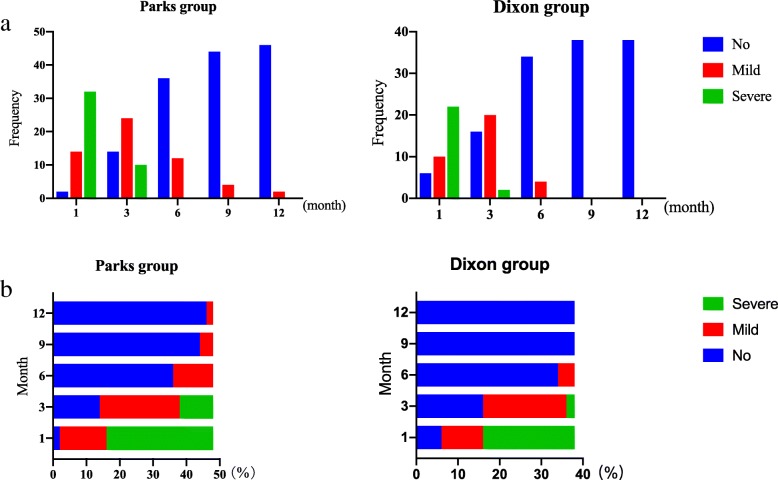
LARS score. **a** Frequency distribution of LARS score in the Parks group and Dixon group. **b** The rate of non, mild, and severe LARS in the Parks group and Dixon group. The total score is 42 points, 0–20 is no LARS; 21–29 is mild LARS; 30–42 is severe LARS

From 1 to 9 months after surgery, the LARS score in the Parks group was more serious (P1 = 0.001, P3 = 0.014, P6 = 0.022, P9 = 0.042, LARS rate 95.25–70.83–25.00–8.33% vs 84.21–57.89–10.53–0.00%). However, the LARS score in both groups was decreased month by month. Until 12 months after surgery, the LARS score in both groups was similar and improved significantly (*P* = 0.065, LARS rate 4.17% vs 0.00%) (Figs. 4b and 6).

##### Wexner scale

From 1 to 6 months after surgery, the Wexner score of the Parks group was higher (P1 = 0.000, P3 = 0.001, P6 = 0.039), but the Wexner score of both groups was decreasing. From 9 to 12 months after surgery, the Wexner score in the two groups was similar (P9 = 0.054, P12 = 0.075) and improved significantly (Fig. 6).

#### FIQL scale

##### Lifestyle domain

**Fig. 5:**
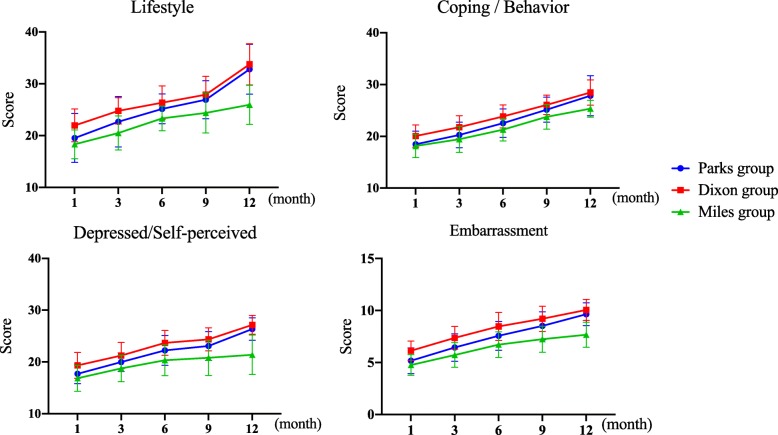
The change trend of FIQL four-domain score after operation in three groups

**Fig. 6:**
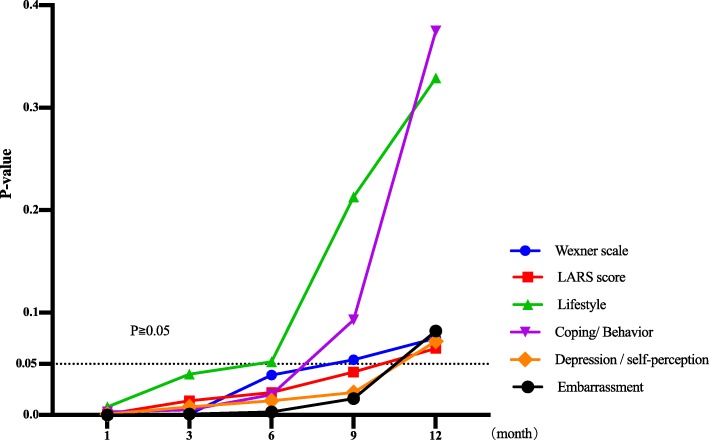
*T* test results of postoperative Wexner scale, LARS score, and FIQL score in Parks-Dixon group

From 1 to 3 months after surgery, the score of the Parks group was lower (P1 = 0.008, P3 = 0.040). However, from 6 to 12 months after surgery, the scores of the two groups were similar (P6 = 0.052, P9 = 0.213, P12 = 0.329) and gradually increased (Figs. 5 and 6).

##### Coping/behavior domain

From 1 to 6 months after surgery, the scores of the Parks group were lower (P1 = 0.003, P3 = 0.005, P6 = 0.020). However, from 9 to 12 months after surgery, the scores of the two groups were similar (P9 = 0.093, P12 = 0.375) and increased month by month (Figs. 5 and 6).

##### Depression/self-perception domain and the embarrassment domain

From 1 to 9 months after surgery, the scores of two domains in the Parks group were lower (P1 = 0.001/0.000, P3 = 0.008/0.001, P6 = 0.014/0.003, P9 = 0.022/0.016). It was not until 12 months after surgery that these two domain scores of the two groups were similar (P12 = 0.072/0.082) (Fig. 6).

### Parks-Miles group

#### FIQL score

##### Lifestyle domain

**Fig. 7:**
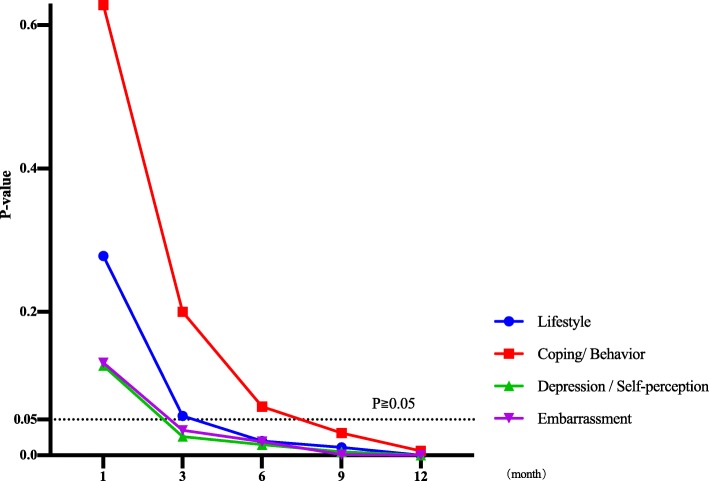
*T* test results of postoperative FIQL score in Parks-Miles group

From 1 to 3 months after surgery, the scores of the two groups were similar (P1 = 0.278, P3 = 0.055). However, from 6 to 12 months after surgery, the scores of the Parks group were higher (P6 = 0.020, P9 = 0.011, P12 = 0.000), and the score gap increased gradually (Figs. 5 and 7).

##### Coping/behavior domain

From 1 to 6 months after surgery, the scores of the two groups were similar (P1 = 0.628, P3 = 0.200, P6 = 0.068). However, from 9 to 12 months after surgery, the score of the Parks group was higher (P9 = 0.031, P12 = 0.006), and the score gap increased gradually (Figs. 5 and 7).

##### Depression/self-perception domain and embarrassment domain

From 1 month after surgery, the scores of the two groups were similar (P1 = 0.125/0.192). However, from 3 to 12 months after surgery, the scores of the Parks group were higher in these two domains (P3 = 0.026/0.035, P6 = 0.015/0.019, P9 = 0.005/0.001, P12 = 0.000/0.000), and the score gap increased gradually (Figs. 5 and 7).

## Discussion

At present, there are four types of anus-preserving operation for ultra-low rectal cancer: ultra-low anterior resection (Dixon operation), intersphincter resection (ISR operation) [27], coloanal anastomosis (Parks operation), and local transanal resection. Among them, ISR [28] is a relatively mature anus-preserving operation, but because it needs to remove part or all of the internal anal sphincter, the postoperative anal function of patients is not good [29]. Therefore, the research on other more effective anus-preserving methods has never stopped.

Parks operation is a kind of anus-preserving operation put forward by Parks in 1982. However, because of its open operation, the operation is difficult, the patients have many complications, and the anal function is not good. Nowadays, the application of laparoscopic technology makes up for the shortcomings of traditional Parks operation, and the application of laparoscopic Parks operation in anus-preserving operation for ultra-low rectal cancer is increasing gradually. Denost et al. [30] also confirmed the oncological safety and effectiveness of laparoscopic Parks procedure for the extraction of anal tumors. However, there are few reports on anal function and quality of life related to anal function in patients who underwent laparoscopic Parks surgery. By combining the advantages of traditional Bacon operation [25, 26], our treatment group adopted improved Parks operation under laparoscopy for anus-preserving of ultra-low rectal cancer patients, which achieved rapid recovery and good anal function, and met the requirements of many patients without incision, minimally invasive and beautiful. This operation method is an important innovation for ultra-low rectal cancer, which is worthy of clinical popularization.

### Basic information

Compared with the Dixon group, the patients in the Parks group were more obese (*P* ≤ 0.030), the tumor was closer to the anal margin (*P* ≤ 0.000), but the total cost of hospitalization was lower (*P* ≤ 0.000). Compared with the Miles group, the body type (*P* ≤ 0.366) and the distance between the tumor and the anal margin (*P* ≤ 0.102) were similar in the two groups, but the hospitalization cost in the Parks group was less (*P* ≤ 0.016) (Table 2).

The follow-up period of this study was 12 months after surgery, and no operative death occurred. The following is a discussion of the results of the anal function and FIQL scale.

### Anal function

On the premise of ensuring radical treatment, compared with Dixon group, the position of tumor in the Parks group was lower, the rectal and anal canal was inevitably retained less, and the anal sphincter and dentate line were more damaged, so the early anal function of the Parks group was worse. With the regular anal function intensive exercise, 6 months after surgery, the anal function of the two groups was similar (Williams classification standard, Wexner scale). However, the LARS score of the two groups was not close until 12 months after surgery, suggesting that fecal incontinence recovered quickly in the Parks group, but there was still a long-term and more serious symptom of anterior rectal resection.

#### FIQL scale

##### Parks-Dixon group

Compared with the Dixon group, the scores of early four domains in Parks group were lower, indicating that early postoperative fecal incontinence had a greater negative impact on the quality of life of patients. However, from 6 to 12 months after surgery, the gap between the four domains of the two groups gradually narrowed and reached a similar level successively.

Lifestyle domain: We believe that the change of lifestyle may be mainly related to the regular exercise of anal function after surgery and the gradual approach to normal anal function. The transition from having to use a pad, moderate diet, and fear of going out to getting rid of the restraint of pad and diet has also greatly improved the quality of life in the domain of lifestyle after surgery.

Coping/behavior domain: The coping/behavior domain did not improve to the same level as that in the Dixon group until 9 months after surgery, which indicated a greater negative impact on this domain of fecal incontinence. We believe that this may be related to the frequent fecal incontinence, which makes patients have to repeatedly enter and out the toilet to defecate. After 6 months, the pad did not need to be used again, but the presence of slight fecal fistula also makes patients still rely on toilet defecation, so the improvement of coping/behavioral domain was relatively slow.

Depression/self-perception domain and the embarrassment domain: It was not until 12 months after surgery that the quality of life in these two domains did improve to the same level as that in the Dixon group. We believe that the slow recovery of the Parks group in these two domains may be related to the following factors: (1) Because of the lower mass location, the patients in Parks group were more worried about their own oncology prognosis after anus preservation. (2) Repeated fecal incontinence after surgery makes the patient think that he is not a healthy person for a long time. Even though the anal function gradually recovers later, the patients were still worried about the repetition of fecal incontinence. (3) The effect of six to eight times postoperative chemotherapy on the family financial burden and the side effects of chemotherapy. These factors make patients feel depressed and embarrassed for a long time after surgery. Until nearly 1 year after surgery, patients gradually adapted to their new rectal function, the anal function was nearly normal, and the postoperative chemotherapy cycle was basically over. The reexamination results of 1 year after surgery also gave patients more hope for their future life, so the depression and embarrassment of patients were better than before.

These results suggest that the patients in the Parks group undergo a gradual recovery process from physiological to psychological. The scores of depression/self-perception and embarrassment were closely related to patients’ cognition of their condition [31]. Therefore, we believe that, in addition to guiding patients with regular anal function exercise, it is also important to give patients more humanistic care, help them to strengthen their understanding [32] and acceptance [19] of their own condition, and establish a more positive and optimistic attitude toward diagnosis and treatment. These will be more conducive to improving the overall quality of life of patients after surgery. This also reflects the biological-psychological-social medical model [33].

##### Parks-miles group

Compared with the Miles group, the FIQL of the two groups was similar in the early postoperative stage. However, 3 months after surgery, the difference of four-domain scores between the two groups gradually increased, and there were significant differences successively.

These results show that anus-preserving advantages of Parks surgery have greatly improved the quality of life of patients after surgery, which the Miles surgery cannot match. This is also consistent with the results reported by Digennaro et al. [34].

There are still some limitations in this study. First of all, the patients with ultra-low rectal cancer in this study did not randomly adopt the above three groups of surgeries. In fact, for tumors with higher position, ultra-low Dixon is more likely to be selected, while for more advanced tumors, APR is preferred, while for tumors with lower stage, Parks is preferred for tumors with earlier stages. This also led to the difference of some preoperative data of the three groups, but by strictly screening the preoperative data of each group, we removed some of the patients with obvious differences and minimized these differences. Secondly, the sample size of this study is relatively small, which may affect the reliability of our conclusions. Thirdly, all the surgeries in this study were performed by the same surgical group and the same doctor. It controls the variables; however, there may be differences in surgical techniques between different doctors. Thus, a multi-center research was needed to further validate our conclusions. Fourthly, this study only followed up the anal function and FIQL of the patients 12 months after surgery, and the oncology prognosis and postoperative intestinal adhesion of the patients for a longer period of time was still lacking. Fifthly, in this study, we give a variety of guidance on postoperative anal functional rehabilitation exercise for patients with anal preservation and control the consistency of the guidance as much as possible. But different patients have different degrees of implementation of the same rehabilitation exercise, and the exercise effect is not the same, which may have an impact on the results of the study. Moreover, we have less guidance on patients’ cognition of postoperative diseases, which may be related to the slow recovery of patients’ cognitive quality of life. For these reasons, large-scale multi-center prospective randomized controlled trials will be carried out in the future to give patients more cognitive guidance on disease and to further study the relationship between the long-term oncological prognosis of these three surgical methods.

## Conclusions

This study shows that laparoscopic modified Parks surgery is a safe and effective anus-preserving method for patients whose sphincters have not been invaded and cannot be safely resected and anastomosed through anterior resection. The limitation of Dixon surgery in free distal rectum was overcome by the method of laparoscopic downstream detachment combined with transanal bottom-up detachment [35]. Laparoscopic modified Parks surgery has obvious technical advantages for obese patients and male patients with narrow pelvis because it does not need to anastomose in the pelvis. The faster recovery and fewer complications after surgery are in line with the concept of fast recovery surgery [36]. Moreover, there were no auxiliary incisions in the abdomen, and only four small punctures were left, which not only met the cosmetic needs of the patients, but also avoided the complications related to abdominal incisions, which was also in line with the concept of injury control surgery [37]. In addition, it can also be classified as a kind of NOTES surgery [38].

## Data Availability

The datasets used and analyzed during the current study are available from the corresponding author on reasonable request.

## Abbreviations

APR: Abdominal and perineal resection
FIQL: Fecal incontinence quality of life
ISR: Intersphincteric resection
LARS: Low anterior resection syndrome
NOTES: Nature orifice transluminal endoscopic surgery
TME: Total mesorectal excision
